# Comparing Amniotic Membranes to Other Bioengineered Skin Substitutes in Wound Healing: A Propensity Score-Matched Analysis

**DOI:** 10.3390/jcm14124272

**Published:** 2025-06-16

**Authors:** Micaela J. Tobin, Audrey K. Mustoe, Sasha Nickman, Tricia Mae Raquepo, Mohammed Yamin, Agustin N. Posso, Sarah J. Karinja, Bernard T. Lee, Ryan P. Cauley

**Affiliations:** Division of Plastic and Reconstructive Surgery, Beth Israel Deaconess Medical Center, Harvard Medical School, Boston, MA 02215, USA; mtobin2@bidmc.harvard.edu (M.J.T.); amustoe@bidmc.harvard (A.K.M.); sashan@bu.edu (S.N.); traquepo@bidmc.harvard.edu (T.M.R.); myamin1@bidmc.harvard.edu (M.Y.); aposso@bidmc.harvard.edu (A.N.P.); skarinja@bidmc.harvard.edu (S.J.K.); blee3@bidmc.harvard.edu (B.T.L.)

**Keywords:** amniotic membrane, skin substitute, wound healing, tissue regeneration, burns, chronic wounds

## Abstract

**Background/Objectives**: The amniotic membrane, which is widely available and inexpensive, has received recent attention for its potential applications in wound healing. This is the first study to use a large database to examine the efficacy of amniotic membrane grafting compared to other skin substitutes. **Methods**: The TriNetX electronic health database was queried in October 2024 for patients with burns or chronic skin ulcers. Patients were stratified by treatment with amniotic membrane grafts or another skin substitute. These patients were then 1:1 propensity score-matched based on age, demographics, and comorbidities. Group differences were assessed with risk ratios and *p*-values. **Results**: A total of 557 patients remained in each group after propensity score matching. Patients who were treated with amniotic membrane grafts had significantly decreased hypertrophic scarring (1.7% vs. 6.2%, *p* < 0.0001), local skin infections (17.4% vs. 29.9%, *p* < 0.0001), and acute postoperative pain (3.7% vs. 7.8%, *p* = 0.003). Additionally, subsequent split-thickness skin grafting was utilized significantly less after amniotic membrane grafts. When compared to skin substitutes for large wounds (>100 cm^2^), the advantages of amniotic membrane were even more pronounced. **Conclusions**: This multi-institutional study supports amniotic membranes as a viable alternative to conventional bioengineered skin substitutes. Further research should evaluate amniotic membranes in wound beds of different sizes to better characterize their use in preparation for or as an alternative to skin grafting itself.

## 1. Introduction

Chronic wounds represent a growing challenge to healthcare systems worldwide, affecting millions of patients and consuming substantial healthcare resources. With the aging population and increasing prevalence of diabetes and vascular diseases, these wounds have become more common and more difficult to manage effectively [1]. In the current healthcare landscape, practitioners face mounting pressure to identify cost-effective and clinically superior treatment options for these complex cases. In fact, lower extremity wounds alone affect between 2.4 and 4.5 million people in the United States, often lasting for years and recurring in up to 70% of patients [2,3]. The management of chronic wounds requires frequent wound care and monitoring, and patients face ongoing risks of reinjury, infection, and limb loss. These risks translate to a considerable financial burden on the healthcare system, with wound management consuming approximately 2–3% of total healthcare costs in the United States and representing billions of dollars annually that could be optimized through more effective treatment approaches [4].

The treatment approach for chronic wounds is multifaceted and highly dependent on many clinical factors that must be carefully evaluated when determining the optimal management strategy. These considerations include wound depth, size, location, and infection risk. Traditional management strategies include debridement, negative pressure wound therapy, compression, and split-thickness skin grafting, the last of which remains the gold standard for traumatic skin loss [4,5,6,7]. While these approaches have demonstrated efficacy in many cases, split-thickness skin grafting presents several notable constraints, chief among them being the requirement for available donor skin, which may be limited in patients with extensive wounds or compromised skin integrity [5]. This has driven the development of alternative solutions aimed at overcoming the challenges posed by conventional approaches [4,8]. These include bioengineered skin substitutes, which are frequently used to prepare unsuitable wound beds without adequate vascularity for eventual skin grafting. Since the development of the first artificial dermal substitute in the 1980s (the Integra Dermal Regeneration Template), the market has expanded dramatically [9]. There are now approximately 76 available skin substitutes, classified according to structure and composition [10].

One particularly promising advancement in the field of skin substitutes is the amniotic membrane, which comprises the innermost layer of the placenta and demonstrates a complex structure consisting of a single epithelial layer, a basement membrane, and an avascular stroma. This specialized tissue is ethically sourced from consenting individuals during planned cesarean sections, after which it undergoes careful sterilization and processing techniques designed to preserve its valuable extracellular matrix components and growth factor profile while ensuring safety for clinical application [11,12,13]. The therapeutic potential stems from its inclusion of growth factors and cytokines that stimulate cell proliferation and migration within the wound bed [14]. Additionally, the membrane possesses notable anti-inflammatory properties that contribute to reduced scarring and improved aesthetic outcomes, along with antibacterial characteristics that help prevent infection [14]. It is these properties that have gained the recent attention of popular news outlets, generating increased interest in amniotic membrane applications across both medical professionals and patients [15].

In the context of treating wounds, early clinical evidence regarding amniotic membrane applications has been promising. One case series of seven patients who failed conventional therapies showed complete healing in six of these patients within eight weeks of amniotic membrane application [16]. Another randomized control trial achieved a 92% healing rate after 6 weeks in those treated with amniotic membranes for indolent diabetic foot ulcers compared to an 8% healing rate in the standard treatment group [17]. Despite these encouraging preliminary results, it is important to acknowledge that the majority of existing evidence comes from relatively small case series and fundamental basic science research rather than large-scale clinical trials [18,19,20,21]. While these studies have provided foundational knowledge about the mechanisms through which amniotic membranes exert their effects, this limitation can be attributed to several significant challenges inherent to researching amniotic membrane applications, including considerable variability in amniotic membrane preparation methods, heterogeneity in wound types and characteristics, and the substantial complexity involved in standardizing treatment protocols across diverse clinical settings [22]. Consequently, the current evidence base remains somewhat limited in both scale and scope, potentially affecting the generalizability of findings and making it difficult for clinicians to draw definitive conclusions regarding the broader application of amniotic membranes across varied patient populations and wound types [21].

This study addresses this knowledge gap by evaluating the therapeutic efficacy of amniotic membrane applications in wound healing through a large-scale multi-institutional database analysis. By leveraging the TriNetX electronic health database, which contains de-identified records from 97 healthcare organizations and over 130 million patients, this study offers a unique opportunity to assess outcomes across diverse patient populations and clinical settings. Through propensity score matching, we seek to provide evidence to guide decision making regarding the application of amniotic membranes in wound management.

## 2. Materials and Methods

A retrospective cohort study was conducted in the TriNetX electronic health database (Cambridge, MA, USA) in October 2024. TriNetX is a health research network that aggregates de-identified health records from 97 healthcare organizations across the United States, providing access to clinical data from over 130 million patients. This database provides real-time access to structured electronic health data, including diagnoses, procedures, medications, laboratory results, and demographic information. The Institutional Review Board (IRB) at our hospital determined that this study did not constitute human subject research as it only utilized de-identified data (IRB protocol #: 2024D000940).

Two primary cohorts were defined for analysis. The exposure cohort consisted of patients who received amniotic membrane grafts for burns or chronic skin ulcers, and the control cohort included patients who received alternative bioengineered skin substitutes for similar indications. The patients were identified with specific procedure codes for amniotic membrane application and other bioengineered skin substitute placement. Data extraction relied on Current Procedural Terminology (CPT), International Classification of Diseases, Tenth Revision (ICD-10), and Healthcare Common Procedure Coding System (HCPCS) codes (Table A1).

The inclusion criteria for both cohorts comprised patients with documented diagnoses of burns or chronic skin ulcers who received treatment with either amniotic membrane grafts or other bioengineered skin substitutes and had available follow-up data. Patients were excluded if they received a combination of different types of skin substitutes.

To minimize selection bias and confounding variables, propensity score matching with logistic regression was performed using the TriNetX platform’s built-in logistic regression algorithm. Patients were 1:1 matched. The matching variables included demographics (age, sex, race, and ethnicity) and comorbidities known to affect wound healing (diabetes mellitus, peripheral vascular disease, obesity, and smoking status). The quality of matching was assessed by examining standardized differences between the matched groups for each covariate.

All outcomes were assessed at one year following the index procedure through an analysis of diagnostic codes entered into the electronic health record. The primary outcomes were hypertrophic scarring and local skin infections. Secondary outcomes included graft-related complications, wound dehiscence, graft failure, requirement for subsequent split-thickness skin grafting, and acute postoperative pain.

To evaluate the comparative effectiveness of amniotic membrane grafts across different wound sizes, a subgroup analysis was conducted. This analysis compared amniotic membrane grafts of any size to bioengineered skin substitutes specifically used for smaller wounds (<25 cm^2^) and to bioengineered skin substitutes specifically used for larger wounds (>100 cm^2^). The wound size classification for bioengineered skin substitutes was determined using CPT codes that specify the wound area treated. The same propensity score matching approach was used for these subgroup analyses to ensure comparable cohorts.

All statistical analyses were conducted within the TriNetX analytics platform. For all outcomes, the absolute risk in each cohort, risk ratios (RRs) with 95% confidence intervals, and corresponding *p*-values were calculated, with *p* ≤ 0.05 considered statistically significant. Categorical variables were analyzed using the chi-squared test to assess differences in proportions between groups. Continuous variables, such as age, were analyzed using Student’s *t*-tests to assess differences in means between groups. To protect patient privacy in accordance with TriNetX protocols, cell counts less than or equal to 10 were suppressed and reported as “≤10” in the results.

## 3. Results

After querying the TriNetX database, 593 patients who received amniotic membrane grafts for burns or chronic skin ulcers were identified, and 43,583 patients were treated with alternative bioengineered skin substitutes of any size. Before matching, significant differences were observed between the cohorts, including age at index (61.3 vs. 58.8 years, *p* = 0.001), ethnicity (Non-Hispanic: 57.3% vs. 72.9%, *p* < 0.001), peripheral vascular disease (19.4% vs. 29.0%, *p* < 0.001), and type 2 diabetes (38.4% vs. 44.7%, *p* = 0.002) (Table 1).

After 1:1 propensity score matching, 593 patients remained in each cohort. The matched groups showed no significant differences in demographic and clinical characteristics, including age (61.3 vs. 61.8 years, *p* = 0.591), race (White: 67.5% in both groups, *p* = 1.0), ethnicity (Non-Hispanic: 57.3% vs. 57.8%, *p* = 0.86), sex (male: 58.9% vs. 59.4%, *p* = 0.859), peripheral vascular disease (19.4% vs. 20.7%, *p* = 0.562), type 1 diabetes (9.8% vs. 8.9%, *p* = 0.618), and type 2 diabetes (38.4% vs. 39.3%, *p* = 0.766) (Table 1). Matching was similarly effective in the propensity score matching for subset analyses.

In the matched cohorts, patients treated with amniotic membrane grafts demonstrated significantly lower rates of hypertrophic scarring compared to those treated with other bioengineered skin substitutes (1.69% vs. 6.24%, RR 0.27, *p* < 0.0001). Local skin infections, another primary outcome, were also significantly reduced in the amniotic membrane group, occurring in 17.37% of these patients compared to 29.85% in the skin substitute group (RR 0.58, *p* < 0.0001) (Table 2).

Regarding secondary outcomes, the amniotic membrane group showed reduced rates of wound dehiscence (4.89% vs. 7.76%, RR 0.63, *p* = 0.04), and acute postoperative pain was reported in 3.71% of patients in the amniotic membrane group compared to 7.76% in the skin substitute group (RR 0.48, *p* = 0.003). Patients in the amniotic membrane group also had significantly lower rates of subsequent split-thickness skin grafting (STSG) (1.69% vs. 13.15%, RR 0.13, *p* < 0.0001). The occurrence of graft complications was also lower in the amniotic membrane group, but did not reach statistical significance (2.19% vs. 4.05%, RR 0.54, *p* = 0.06). Graft failure rates were comparable between the two groups (1.69% vs. 2.19%, RR 0.77, *p* = 0.53) (Table 2).

When comparing amniotic membrane grafts to skin substitutes used for larger wounds (>100 cm^2^), 591 matched patient pairs were analyzed. In this comparison, amniotic membrane grafts were associated with significantly lower rates of hypertrophic scarring (1.69% vs. 13.71%, RR 0.12, *p* < 0.0001), graft complications (2.20% vs. 7.28%, RR 0.30, *p* < 0.0001), and subsequent STSG (1.69% vs. 26.23%, RR 0.07, 95%, *p* < 0.0001). Additionally, patients treated with amniotic membrane grafts had lower rates of local skin infections (*p* = 0.02), graft failure (*p* = 0.01), and acute postoperative pain (3.72% vs. 13.37%, RR 0.28, *p* < 0.0001) compared to those receiving bioengineered skin substitutes for large wounds (Table 2). There was no significant difference in wound dehiscence rates (4.91% vs. 6.94%, RR 0.70, *p* = 0.14).

When comparing amniotic membrane grafts to skin substitutes used for smaller wounds (<25 cm^2^), 590 matched patient pairs were analyzed. In this analysis, amniotic membrane grafts were associated with significantly lower rates of wound dehiscence (4.92% vs. 8.81%, RR 0.56, *p* = 0.008), subsequent STSG (1.69% vs. 4.41%, *p* = 0.007), and local skin infections (17.46% vs. 35.09%, RR 0.49, *p* < 0.0001). However, there were no significant differences in hypertrophic scarring (1.69% vs. 1.69%, *p* = 1.0), graft complications (2.20% vs. 2.20%, *p* = 1.0), graft failure (1.69% vs. 1.69%, *p* = 1.0), or acute postoperative pain (3.73% vs. 5.09%, RR 0.73, *p* = 0.26) (Table 2).

## 4. Discussion

This large-scale, propensity score-matched analysis provides evidence that amniotic membrane grafts represent a viable alternative to conventional bioengineered skin substitutes in treating burns and chronic skin ulcers. Amniotic membrane grafts may offer comparable or superior outcomes across multiple parameters, including hypertrophic scarring, wound dehiscence, local skin infections, and acute postoperative pain. These advantages became particularly apparent when compared to bioengineered skin substitutes used for larger wounds (>100 cm^2^). However, more prospective research will be needed to determine which wounds are most appropriate for amniotic membrane treatment compared to the placement of a skin substitute.

Many characteristics are known to affect wound healing, including those controlled for by propensity score matching, such as tobacco use, peripheral vascular disease, and diabetes. Tobacco use does so through several mechanisms, including the vasoconstrictive effects of nicotine, which reduces blood flow and oxygen delivery to the wound site, causes inflammatory response disarray, and downregulates collagen synthesis and deposition [23,24]. Vascular disease similarly limits perfusion to the distal extremities and reduces the delivery of oxygen and nutrients that are essential for wound repair [25]. In addition to its effects on microcirculation, hyperglycemia, which is a hallmark of diabetes, leads to chronic inflammation, immune dysfunction, and vascular damage, which collectively impede wound healing [26]. While these characteristics are the same across both type 1 and 2 diabetes, the mechanisms are distinct in that type 1 diabetes does so through systemic low-grade inflammation and leukotrienes, and type 2 diabetes does so through epigenetic changes and impaired angiogenesis [27,28,29,30]. By propensity score matching for each of these factors, consistency was maintained between groups in order to better isolate the effects of amniotic membranes alone.

Of note, there is much legislation in the United States that addresses the use of amniotic membranes in the context of wound healing. Specifically, the U.S. Food and Drug Administration (FDA) regulates amniotic membrane products under the Human Cells, Tissues, and Cellular and Tissue-Based Products (HCT/Ps) framework [31]. This regulation ensures that amniotic membrane products meet safety and efficacy standards before they can be marketed and used in clinical settings. This FDA oversight also includes requirements about donor eligibility, processing, labeling, and distribution of amniotic membrane products, which are designed to prevent the transmission of communicable diseases and ensure that products are safe.

When considering sterilization and the preservation of the amniotic membrane, several methods can be used. For sterilization, antibiotic-based aseptic decontamination with low-temperature vacuum-drying has been shown to effectively remove any microbial burden while maintaining antibacterial activity [32]. Supercritical carbon dioxide combined with peracetic acid is another method that produces sterilization while preserving its properties [33]. Cryopreservation is then commonly conducted at −80 degrees Celsius, or freeze-drying can be used to allow for storage at room temperature with a shelf life of up to 20 months [34]. Similar to conventional membranes, the amniotic membrane can then be used and secured with sutures, fibrin glue, or other adhesives. The specific attachment method may influence graft integration and stability, particularly in challenging wound locations or those subject to mechanical stress.

In the context of wound healing outcomes, when investigating the effect on hypertrophic scarring, the markedly lower rates in the amniotic membrane group substantiate previous studies on the anti-fibrotic properties of amniotic tissue. These properties are believed to derive from secretory factors released by amniotic membrane epithelial and mesenchymal stem cells [35]. Mechanistically, these factors inhibit TGF-β signaling and myofibroblast differentiation to subsequently mitigate hypertrophic scar formation [35]. More specifically, the amniotic membrane has been shown to suppress TGF-β1 and TGF-β2 signaling, which are known profibrotic cytokines, while promoting TGF-β3 expression, which has anti-scarring properties [35]. This modulation of the TGF-β pathway represents an important mechanism through which amniotic membranes may reduce excessive scarring.

Additional antifibrotic properties may derive from the ability of amniocytes to modulate the anti-inflammatory M2 macrophages, facilitating the transition from the inflammatory to the tissue repair phase of wound healing [36]. This immunomodulatory effect represents a key advantage of amniotic membrane over synthetic substitutes, as it can actively influence the wound microenvironment rather than serve as a passive scaffold for regeneration.

The significant reductions we observed in wound dehiscence rates and need for subsequent split-thickness skin grafting suggest enhanced wound stability and healing. This improved healing may stem from the membrane’s composition with over 200 regulatory proteins, growth factors, cytokines, and proteases that regulate tissue repair [37]. Key growth factors identified in amniotic membrane include the epidermal growth factor (EGF), keratinocyte growth factor (KGF), hepatocyte growth factor (HGF), basic fibroblast growth factor (bFGF), transforming growth factor alpha and beta (TGF-α and TGF-β), and vascular endothelial growth factor (VEGF) [20]. These factors collectively promote re-epithelialization, angiogenesis, and extracellular matrix remodeling, which are all essential processes for effective wound closure and stability. Furthermore, the basement membrane side of the amniotic membrane contains collagen types IV, V, and VII; laminin; and fibronectin, which provide structural support and serve as a substrate for epithelial cell attachment and migration [38]. The membrane’s physical properties also contribute to wound healing by maintaining a moist wound environment while allowing for gas exchange and preventing fluid loss, conditions known to be optimal for epithelialization [39].

However, it remains unclear whether the observed reduced rate of split-thickness skin grafting is secondary to decreased clinical need or simply represents a planned approach where bioengineered skin substitutes are commonly used as preparatory materials. This distinction could highlight the potential for amniotic membranes to serve as temporary coverings and also as definitive treatments that may obviate the need for subsequent procedures.

The lower rates of local skin infections in the amniotic membrane group support the antimicrobial properties that have been attributed to amniotic tissue in previous research. Specifically, it has been demonstrated that the amniotic membrane exhibits broad-spectrum antimicrobial activity against common wound pathogens, including *Staphylococcus aureus*, *Pseudomonas aeruginosa*, and *Escherichia coli* [35]. The antimicrobial peptides present in the amniotic membrane, including β-defensins and elafin, likely contribute to this protective effect [36]. Additionally, the amniotic membrane has been shown to secrete lysozyme, which degrades bacterial cell walls, particularly those of Gram-positive bacteria [40]. This intrinsic antimicrobial activity provides an advantage over synthetic substitutes that often require the addition of antimicrobial agents. Beyond direct antimicrobial effects, the ability of amniotic membranes to reduce the local immune response by enhancing macrophage and neutrophil activity and limiting excessive inflammation is likely an additional contributory factor to aid in infection control [36,41].

Finally, the significant reduction in acute postoperative pain in the amniotic membrane group also represents an important advantage of these grafts. The pain-reducing effects of amniotic membrane can be attributed to several mechanisms. First, the membrane provides a barrier that covers exposed nerve endings in the wound bed, shielding them from external stimuli [42,43]. Second, the anti-inflammatory properties of the membrane reduce the concentration of inflammatory mediators such as prostaglandins, bradykinin, and histamine, which are known to sensitize nociceptors and contribute to pain sensation [44]. Additionally, the neurotrophic stem cells secreted from amniotic membrane-derived mesenchymal stem cells have been shown to secrete neurotrophic factors that shield exposed nerve endings in wounds [45,46]. These include nerve growth factor (NGF), brain-derived neurotrophic factor (BDNF), and glial cell line-derived neurotrophic factor (GDNF), which not only protect neurons but may promote nerve regeneration in chronic wounds.

Subgroup analyses revealed that the benefits of amniotic membranes may be particularly pronounced when compared to bioengineered skin substitutes used for larger wounds. In these comparisons, amniotic membrane grafts were associated with dramatically lower rates of hypertrophic scarring and subsequent split-thickness skin grafts. Given that the size of wounds that are covered with amniotic membranes is unknown, it is possible that this difference could be attributed to the amnion’s use in smaller or less complex wounds with inherently better healing potential. Yet, a differential effect in larger wounds is supported by previous work by other authors, which demonstrates that amniotic membranes’ anti-inflammatory and pro-angiogenic effects become increasingly valuable as wound complexity increases [47]. The superior performance in larger wounds may be attributed to several properties, including the sustained release of growth factors and matrix proteins, more rapid epithelialization at wound edges, and granulation tissue formation across large surface areas [47].

Additionally, the inflammatory response in larger wounds is typically more pronounced and prolonged, potentially leading to excessive scarring and delayed healing [48]. The immunomodulatory properties of amniotic membrane may be particularly beneficial in this context, helping to regulate the inflammatory cascade and prevent its detrimental effects. Additionally, large wounds often face challenges in establishing adequate vascularization throughout the wound bed [49]. The pro-angiogenic factors in the amniotic membrane, including VEGF and bFGF, may help overcome this challenge by promoting blood vessel formation from the wound periphery.

An important topic not fully explored in this analysis is the cost-effectiveness of amniotic membrane grafts compared to conventional bioengineered skin substitutes. Amniotic membranes are generally less expensive to procure and process than many synthetic or bioengineered alternatives, potentially offering substantial cost savings to healthcare systems [18]. Beyond the direct cost of the graft material, the reduced rates of complications and subsequent procedures associated with amniotic membrane use could translate to significant additional savings. The lower rates of infection, dehiscence, and need for split-thickness skin grafting observed in our study suggest potential reductions in hospital readmissions, additional surgeries, and prolonged wound care [50]. For example, the prevention of a single surgical site infection has been estimated to save between USD 10,000 and USD 25,000 in healthcare costs [51]. From a broader healthcare economics perspective, the use of amniotic membranes may represent an efficient allocation of resources, particularly in healthcare systems with limited access to advanced wound care products or surgical capabilities. Their relatively simple storage requirements and application techniques make them accessible in diverse clinical settings.

Despite our promising findings, several limitations warrant consideration. First, as a retrospective database analysis, this study is unable to account for clinical decision making that led providers to choose amniotic membrane grafts compared to other bioengineered skin substitutes. The observed differences in outcomes between treatment groups, and particularly the lower rates of subsequent split-thickness skin grafting in the amniotic membrane group, raise the possibility that underlying wound severity, rather than graft material alone, may have influenced these outcomes. The database also lacks information about the specific amniotic membrane products used, their processing methods, and application techniques, all of which could influence clinical outcomes. Different preservation methods (cryopreserved, dehydrated, etc.) and orientations (epithelial side up or down) may have different clinical effects that cannot be distinguished in our analysis.

Selection bias is another particular concern, as clinicians may have preferentially used amniotic membranes for wounds with better healing potential. While propensity score matching helps mitigate some of these concerns, it cannot account for unmeasured confounders such as wound characteristics, nutritional status, patient compliance, and local wound care practices. The possibility remains that the observed benefits of amniotic membrane may be partially attributable to their application in more favorable clinical scenarios. This limitation is particularly relevant in the context of the finding that amniotic membranes performed comparably to skin substitutes intended for smaller wounds but significantly outperformed those used in larger wounds. Without additional information about the wounds themselves, it cannot be determined whether amniotic membranes truly demonstrate superior healing properties or whether they were simply applied to less complex cases.

Future research should address these limitations through prospective randomized controlled trials that standardize wound assessment and treatment protocols. These studies should stratify wounds by size, depth, and etiology to determine which wound types benefit most from amniotic membrane application. Standardized assessment tools should be used to document wound healing progression, and outcomes should include patient-reported measures such as pain, function, and quality of life. Cost-effectiveness analyses that account for the broader impact of such treatments could further inform future choices of treatment pathways. These analyses should consider not only direct material costs but also the economic impact of prevented complications, the reduced need for subsequent procedures, and potentially shorter healing times.

## 5. Conclusions

This large-scale, propensity score-matched analysis provides compelling evidence that amniotic membrane grafts represent a viable alternative to conventional bioengineered skin substitutes in the treatment of burns and chronic ulcers. Patients receiving amniotic membrane grafts demonstrated significantly reduced rates of hypertrophic scarring, local skin infections, wound dehiscence, and acute postoperative pain, with an additional decreased need for subsequent split-thickness skin grafting. When compared directly to bioengineered skin substitutes, the advantages were particularly pronounced, suggesting that the anti-inflammatory, antimicrobial, antifibrotic, and pro-angiogenic properties of amniotic tissue may be especially beneficial in more complex wound environments. These findings align with the growing body of translational and clinical research supporting amniotic tissue’s regenerative potential. As the clinical adoption of amniotic membrane technology continues to accelerate, additional prospective, randomized studies with extended follow-up periods are warranted to validate these findings across varied patient populations and wound etiologies. Future research should also focus on defining optimal application protocols, graft preparation techniques, and combination strategies with other advanced wound care modalities.

## Figures and Tables

**Table 1:** Cohort characteristics before and after propensity score matching.

Variable	Before Matching			After Matching		
	Amniotic Membrane (*n* = 593)	All Skin Substitutes (*n* = 43,583)	*p*-Value	Amniotic Membrane (*n* = 593)	All skin substitutes (*n* = 593)	*p*-Value
Age at Index Mean (SD)	61.3 (17.8)	58.8 (17.8)	0.001	61.3 (17.8)	61.8 (17.0)	0.591
White *n* (%)	400 (67.5)	29,547 (67.8)	0.859	400 (67.5)	400 (67.5)	1
Not Hispanic or Latino *n* (%)	340 (57.3)	31,752 (72.9)	<0.001	340 (57.3)	343 (57.8)	0.86
Male *n* (%)	349 (58.9)	26,672 (61.2)	0.244	349 (58.9)	352 (59.4)	0.859
Tobacco use (%)	5.1% (30)	7.1% (3107)	0.051	5.1% (30)	3.9% (23)	0.325
Peripheral vascular disease, unspecified (%)	19.4% (115)	29.0% (12,622)	<0.001	19.4% (115)	20.7% (123)	0.562
Type 1 diabetes mellitus (%)	9.8% (58)	11.8% (5124)	0.137	9.8% (58)	8.9% (53)	0.618
Type 2 diabetes mellitus (%)	38.4% (228)	44.7% (19,470)	0.002	38.4% (228)	39.3% (233)	0.766
	Amniotic Membrane (*n* = 593)	Skin Substitutes for wound areas < 25 cm^2^ (*n* = 23,510)	*p*-Value	Amniotic Membrane (*n* = 590)	Skin Substitutes for wound areas < 25 cm^2^ (*n* = 590)	*p*-Value
Age at Index Mean (SD)	61.3 (17.8)	63.9 (15.3)	<0.0001	61.5 (17.7)	62.1 (16.2)	0.541
White *n* (%)	400 (67.5)	16,823 (71.6)	0.029	400 (67.8)	399 (67.6)	0.950
Not Hispanic or Latino *n* (%)	340 (57.3)	17,760 (75.5)	<0.001	340 (57.6)	344 (58.3)	0.814
Male *n* (%)	349 (58.9)	14,120 (60.1)	0.554	346 (58.6)	349 (59.2)	0.859
Tobacco use (%)	5.1% (30)	7.7% (1805)	0.018	5.1% (30)	4.6% (27)	0.684
Peripheral vascular disease, unspecified (%)	19.4% (115)	38.7% (9093)	<0.0001	19.5% (115)	20.5% (121)	0.662
Type 1 diabetes mellitus (%)	9.8% (58)	15.7% (3701)	<0.0001	9.8% (58)	8.1% (48)	0.309
Type 2 diabetes mellitus (%)	38.4% (228)	56.4% (13,269)	<0.0001	38.6% (228)	37.8% (223)	0.765
	Amniotic Membrane (*n* = 593)	Skin Substitutes for wound areas > 100 cm^2^ (*n* = 12,440)	*p*-Value	Amniotic Membrane (*n* = 591)	Skin Substitutes for wound areas > 100 cm^2^ (*n* = 591)	*p*-Value
Age at Index Mean (SD)	61.3 (17.8)	49.4 (18.6)	<0.0001	61.2 (17.8)	61.5 (17.8)	0.819
White *n* (%)	400 (67.5)	7718 (62.04)	0.008	398 (67.3)	398 (67.3)	1
Not Hispanic or Latino *n* (%)	340 (57.3)	8436 (67.8)	<0.0001	340 (57.5)	355 (60.1)	0.375
Male *n* (%)	349 (58.9)	8017 (64.4)	<0.0001	348 (58.9)	367 (62.1)	0.258
Tobacco use (%)	5.1% (30)	5.4% (674)	0.71	5.1% (30)	4.9% (29)	0.894
Peripheral vascular disease, unspecified (%)	19.4% (115)	9.0% (1115)	<0.0001	19.3% (114)	18.6% (110)	0.767
Type 1 diabetes mellitus (%)	9.8% (58)	3.9% (485)	<0.0001	9.6% (57)	8.1% (48)	0.358
Type 2 diabetes mellitus (%)	38.4% (228)	22.1% (2744)	<0.0001	38.2% (226)	35.4% (209)	0.305

**Table 2:** Risk analysis of 1-year postoperative outcomes in patients who were treated with amniotic membrane grafts vs. other bioengineered grafts.

All Skin Substitutes (*n* = 593)				
	Amniotic membrane *n* (%)	Skin Substitute *n* (%)	Risk Ratio (95% CI)	*p*-Value
Hypertrophic Scarring	≤10 (1.69) *	37 (6.24)	0.27 (0.14, 0.54)	<0.0001
Graft Complications	13 (2.19)	24 (4.05)	0.54 (0.28, 1.05)	0.06
Wound dehiscence	29 (4.89)	46 (7.76)	0.63 (0.40, 0.99)	0.04
Subsequent STSG	≤10 (1.69) *	78 (13.15)	0.13 (0.07, 0.25)	<0.0001
Local Skin Infection	103 (17.37)	177 (29.85)	0.58 (0.47, 0.72)	<0.0001
Graft Failure	≤10 (1.69) *	13 (2.19)	0.77 (0.34, 1.74)	0.53
Acute Postoperative Pain	22 (3.71)	46 (7.76)	0.48 (0.29, 0.79)	0.003
Skin Substitutes for wound areas < 25 cm^2^ (*n* = 590)				
	Amniotic membrane *n* (%)	Skin Substitute *n* (%)	Risk Ratio (95% CI)	*p*-Value
Hypertrophic Scarring	≤10 (1.69) *	≤10 (1.69) *	n/a	1
Graft Complications	13 (2.20)	13 (2.20)	n/a	1
Wound dehiscence	29 (4.92)	52 (8.81)	0.56 (0.36, 0.87)	0.008
Subsequent STSG	≤10 (1.69) *	26 (4.41)	0.39 (0.19, 0.79)	0.007
Local Skin Infection	103 (17.46)	207 (35.09)	0.49 (0.41, 0.61)	<0.0001
Graft Failure	≤10 (1.69) *	≤10 (1.69) *	n/a	1
Acute Postoperative Pain	22 (3.73)	30 (5.09)	0.73 (0.43, 1.26)	0.26
Skin Substitutes for wound areas > 100 cm^2^ (*n* = 591)				
	Amniotic membrane *n* (%)	Skin Substitute *n* (%)	Risk Ratio (95% CI)	*p*-Value
Hypertrophic Scarring	≤10 (1.69) *	81 (13.71)	0.12 (0.07, 0.24)	<0.0001
Graft Complications	13 (2.20)	43 (7.28)	0.3 (0.16, 0.56)	<0.0001
Wound dehiscence	29 (4.91)	41 (6.94)	0.70 (0.45, 1.12)	0.14
Subsequent STSG	≤10 (1.69) *	155 (26.23)	0.07 (0.03, 0.12)	<0.0001
Local Skin Infection	102 (17.26)	133 (22.50)	0.77 (0.61, 0.97)	0.02
Graft Failure	≤10 (1.69) *	24 (4.06)	0.42 (0.20, 0.86)	0.01
Acute Postoperative Pain	22 (3.72)	79 (13.37)	0.28 (0.18, 0.44)	<0.0001

* To protect patient privacy, numbers are rounded up to 10. Abbreviations: split-thickness skin graft (STSG).

## Data Availability

Restrictions apply to the availability of these data. Data were obtained from TriNetX and are available from the authors with the permission of TriNetX.

## Appendix A

**Table A1:** ICD-10, CPT, and HCPCS codes used for data extraction.

**Injury**	
*ICD-10*	*Definition*
L89	Pressure Ulcer
L97	Non-pressure chronic ulcer of lower limb, not elsewhere classified
L98.4	Non-pressure chronic ulcer of skin, not elsewhere classified
T20	Burn and corrosion of head, face, and neck
T21	Burn and corrosion of trunk
T22	Burn and corrosion of shoulder and upper limb, except wrist and hand
T23	Burn and corrosion of wrist and hand
T24	Burn and corrosion of lower limb, except ankle and foot
T25	Burn and corrosion of ankle and foot
**Skin Substitute**	
*CPT*	*Definition*
15271	Application of skin substitute graft to trunk, arms, and legs; total wound surface area up to 100 sq cm, with first 25 sq cm or less wound surface area
15272	Application of skin substitute graft to trunk, arms, and legs; total wound surface area up to 100 sq cm, with each additional 25 sq cm wound surface area or part thereof
15273	Application of skin substitute graft to trunk, arms, and legs; total wound surface area greater than or equal to 100 sq cm, with first 100 sq cm wound surface area, or 1% of body area of infants and children
15274	Application of skin substitute graft to trunk, arms, and legs; total wound surface area greater than or equal to 100 sq cm; each additional 100 sq cm wound surface area, or part thereof, or each additional 1% of body area of infants and children, or part thereof
15275	Application of skin substitute graft to face, scalp, eyelids, mouth, neck, ears, orbits, genitalia, hands, feet, and/or multiple digits; total wound surface area up to 100 sq cm, with first 25 sq cm or less wound surface area
15276	Application of skin substitute graft to face, scalp, eyelids, mouth, neck, ears, orbits, genitalia, hands, feet, and/or multiple digits; total wound surface area up to 100 sq cm, with each additional 25 sq cm wound surface area, or part thereof
15277	Application of skin substitute graft to face, scalp, eyelids, mouth, neck, ears, orbits, genitalia, hands, feet, and/or multiple digits; total wound surface area greater than or equal to 100 sq cm; first 100 sq cm wound surface area, or 1% of body area of infants and children
15278	Application of skin substitute graft to face, scalp, eyelids, mouth, neck, ears, orbits, genitalia, hands, feet, and/or multiple digits; total wound surface area greater than or equal to 100 sq cm; each additional 100 sq cm wound surface area, or part thereof, or each additional 1% of body area of infants and children, or part therof
**Amniotic Membrane**	
*HCPCS*	*Definition*
V2790	Amniotic membrane for surgical reconstruction, per procedure

Abbreviations: Current Procedural Terminology (CPT), International Classification of Diseases, Revision 10 (ICD-10), Healthcare Common Procedure Coding System (HCPCS).
